# Trajectories of Clozapine Concentrations in Women Across Menopausal Age

**DOI:** 10.1093/schbul/sbaf186

**Published:** 2025-10-28

**Authors:** Franciska de Beer, Iris M H Hamers, Michalina Prycka, Georgios Schoretsanitis, Shiral S Gangadin, Daan J Touw, Iris E C Sommer

**Affiliations:** Centre for Clinical Neuroscience and Cognition, University Medical Centre Groningen, University of Groningen, 9713 AP Groningen, The Netherlands; Centre for Clinical Neuroscience and Cognition, University Medical Centre Groningen, University of Groningen, 9713 AP Groningen, The Netherlands; Centre for Clinical Neuroscience and Cognition, University Medical Centre Groningen, University of Groningen, 9713 AP Groningen, The Netherlands; Department of Psychiatry, Psychotherapy and Psychosomatics, Hospital of Psychiatry, University of Zurich, CH 8032 Zurich, Switzerland; Department of Psychiatry, Northwell Health, The Zucker Hillside Hospital, Glen Oaks, NY 11004, United States; Centre for Clinical Neuroscience and Cognition, University Medical Centre Groningen, University of Groningen, 9713 AP Groningen, The Netherlands; Department of Clinical Pharmacy and Pharmacology, University Medical Center Groningen, University of Groningen, 9713 AV Groningen, The Netherlands; Department of Pharmaceutical Analysis, Groningen Research Institute of Pharmacy, University of Groningen, 9700 AD Groningen, The Netherlands; Centre for Clinical Neuroscience and Cognition, University Medical Centre Groningen, University of Groningen, 9713 AP Groningen, The Netherlands

**Keywords:** menopause, clozapine, clozapine pharmacokinetics, CYP1A2

## Abstract

**Background and Hypothesis:**

During menopause, estrogen levels change dramatically, which may decrease clozapine blood concentrations in women via estrogen’s inhibitory effect on CYP1A2 activity. This reduction could contribute to increased relapse rates seen in older women with psychotic disorders.

**Study Design:**

Clozapine blood concentration data were retrieved from the University Medical Center Groningen, the Netherlands. A total of 982 patients (720 men, 262 women), aged 40-60, with 17 104 measurements, were included for analyses. Latent class growth analysis (LCGA) assessed clozapine trajectories by sex, while linear mixed-effects models (LMEM) assessed sex differences between trajectory classes.

**Study Results:**

The optimal LCGA model (7-quantile splines) identified 3 clusters. Most women (*n* = 157, 60%) showed a decline in clozapine levels from 520 to 400 μg/L between the ages of 40-60. In contrast, most men (*n* = 392, 54%) had stable levels (mean 460 μg/L). Two other trajectories appeared in both sexes: a mild increase starting at age 45 (men: *n* = 272, 38%; women: *n* = 97, 37%) and a marked increase from 40 to 60 (men: *n* = 56, ~8%; women: *n* = 8, ~3%). LMEM showed significantly higher levels in women than men with stable trajectories (estimate = 177.03, *t* = 2.62, *P* < .01). A significant age-by-sex interaction (estimate = −0.067, *t* = −2.63, *P* < .01) suggested these differences varied over time.

**Conclusions:**

Sex-specific longitudinal trajectories of clozapine concentrations showed declines in 60% of women aged 40-60, while most men remained stable. As decreasing blood levels could increase relapse vulnerability, monitoring clinical efficacy and side effects is warranted during menopause.

## Introduction

Pharmacokinetic processes show significant sex differences, partly due to estrogen’s influence on drug absorption, distribution, metabolism, and excretion.^1^ These differences are not static but fluctuate in response to hormonal changes throughout a woman’s life. Estrogen levels rise sharply during puberty, fluctuate cyclically throughout the reproductive years, surge during pregnancy, and then drop postpartum. The most pronounced decline occurs during menopause when estrogen levels fall to prepubertal levels, comparable to those observed in age-matched men.^2–4^ Although the impact of exogenous estrogens, such as contraceptives, on the pharmacokinetics of medications has been investigated, the effects of endogenous estrogens remain poorly understood.^5–8^ This knowledge gap should be addressed, as fluctuations in endogenous estrogen may influence pharmacokinetics and, consequently, impact clinically relevant outcomes, including drug efficacy and tolerability.

Clozapine is a second-generation antipsychotic and the only agent approved for treatment-resistant schizophrenia.^9–11^ In Europe, clozapine use ranges from 26-39 per 100 000 women in France to 144-163 per 100 000 women in Finland.^12^ Studies have shown significant sex differences in clozapine blood concentrations when the same doses are prescribed to men and women.^13^^,^^14^ In an extensive therapeutic drug monitoring study, Lane et al. demonstrated that, after controlling for variables such as age and body composition, women had approximately 35% higher clozapine levels compared to age-matched men.^15^ Without correction for body composition, these sex differences are even larger.

Higher clozapine blood concentrations in women when using similar dosages as men are associated with sex differences in the pharmacokinetics for this drug. Women absorb clozapine more rapidly due to lower gastric acidity, exhibit slower gastric emptying, and possess greater fat storage, which prolongs clozapine’s half-life.^16^ They also excrete clozapine more slowly due to lower hepatic and renal blood flow than men.^16^ Together, these factors contribute to higher overall blood levels of clozapine in women.^16^ Furthermore, estrogen influences the hepatic cytochrome P450 (CYP) enzymes responsible for the metabolism of antipsychotics.^16–18^ Estrogen induces the activity of CYP3A4 while it inhibits the activity of CYP1A2 and CYP2C19.^15^^,^^17^ Clozapine is primarily demethylated by CYP1A2 and, to a lesser extent, by CYP3A4 into its metabolite norclozapine.^18^^,^^19^ As a result, the inhibition of CYP1A2 is more pronounced when estrogen levels are high, potentially leading to higher clozapine blood levels. For instance, women using combined oral contraceptives have been shown to require only half the clozapine daily dose prescribed to men to achieve comparable therapeutic drug levels (>300 ng/mL).^18^^,^^19^ The potency of endogenous estrogen as an CYP1A2 inhibitor, and how its inhibition changes during major hormonal transitions, like menopause, remains unclear.

During the menopausal transition, the fluctuating and overall declining estrogen levels may significantly affect clozapine blood concentrations in women. These changes in clozapine levels may impair the efficacy of the antipsychotic, consequently increasing the risk of psychotic relapse in postmenopausal women. Indeed, post-menopausal women have been shown to experience higher relapse rates compared to premenopausal women, but also compared to age-matched men.^20^^,^^21^ However, it is currently unknown if this increase in psychotic disorders is (partly) due to changes in antipsychotic blood levels related to menopause-associated alterations in pharmacokinetics.^5^

Despite these expected pharmacokinetic changes, the trajectories of clozapine blood concentrations in women aged 40-60 remain unstudied. This knowledge gap is particularly concerning given that menopause is already a time of increased vulnerability, further complicated by the physical and psychological burden of menopausal symptoms.^22–25^ Moreover, the lower estrogen levels are thought to affect dopamine neurotransmission, rendering postmenopausal women more vulnerable to relapse.^26^ Therefore, understanding the extent of clozapine’s pharmacokinetic changes during this period is crucial to optimize the treatment outcomes for women using the drug to manage psychotic symptoms. Moreover, as several drugs (eg, olanzapine) have CYP1A2 as their primary metabolizing enzyme in the liver, this study could highlight menopause-related changes in the pharmacokinetics of other drugs.

The present study aimed to identify longitudinal trajectories of clozapine blood concentrations in 982 patients aged 40-60 using latent class growth analysis (LCGA). Trajectories in women were compared with those of men to better understand potential sex-, age-, and menopause-related differences in clozapine pharmacokinetics.

## Methods

### Patients and Samples

Clozapine serum concentrations were collected from the laboratory information system database from the Department of Clinical Pharmacy and Pharmacology of the University Medical Center Groningen (UMCG), the Netherlands. Samples were collected between January 2016 and October 2024 and included serum concentrations, anonymized patient identifier, age, sex, and the date and time of measurement. Only measurements obtained from patients between 40 and 60 years old were included in the analyses, as most women experience menopause during this timeframe.^27^ Data were excluded from the analyses if the patient had fewer than 4 measurements; if attempted suicide by overdose was suspected, identified by multiple serum measurements within 7 days, with at least one exceeding laboratory alert levels; if serum levels exceeded the laboratory alert level of 1000 μg/L^18^^,^^19^; if serum levels were below the lower limit of quantification (LLOQ); if the patient also had a co-medication with carbamazepine, venlafaxine, (nor)fluoxetine, or fluvoxamine within the same month, as these are known to affect clozapine levels,^28–36^ and, in the case of venlafaxine, carries a warning in the Netherlands that it may increase clozapine levels.^29^ The study was registered under the UMCG 21394 registration code. Since the data for our study were obtained from routine healthcare and participation imposed no additional burden on patients, based on Dutch law, our study is not within the reach of medical research for which explicit informed consent is required to use data for scientific purposes. All study procedures adhered to the Declaration of Helsinki (64th WMA General Assembly; October 2013).

### Serum Analyses

Clozapine serum levels were determined in the laboratory of the Department of Clinical Pharmacy and Pharmacology in the UMCG via routine analytical methods (ISO15189). Plasma samples were analyzed with a UPLC-MSMS technique (Thermo Vanquish® and Quantiva® UPLC-MSMS system, equipped with a Thermo Accucore® C18 column, all from Thermo, United States). Samples were prepared by making a mixture of 100 μL plasma and 500 μL of an internal standard solution containing a stable isotope of the drug and 0.04 mg/L cyanoimipramine in methanol, and centrifuging this at 10 000 rpm for 5 min. About 0.5 μL of the supernatant was then injected into the UPLC-MSMS system. A gradient elution method was employed at a flow rate of 1.0 mL/min with an initial mobile phase composition of 0.02 mol/L ammoniumformate buffer (pH 3.5) in Millipore water/methanol (65/35). This ratio transitioned to 5/95 by 1.15 min, followed by re-equilibration to the initial conditions to clean the column for subsequent analyses. The method demonstrated linearity for analyte concentrations ranging from 5 to 500 μL/L, covering the expected serum concentration range (50-450 μg/L). The LLOQ was 5 μg/L, with intra-day and inter-day precision being lower than 15% for low, medium, and high concentrations. All reagents used were of analytical grade. Reference compounds were purchased from Sigma (Netherlands), and stable isotopes were purchased from Alsachim (France).

### Statistical Analysis

To identify trajectories of clozapine concentrations over the years, LCGA was performed separately on longitudinal data of men and women aged 40-60 years. LCGA is a statistical method that classifies individuals with similar longitudinal patterns, also called growth trajectories.^37^ The individual trajectories were modeled with a random intercept representing the initial clozapine level at the first assessment, and a spline fitting method with quantile splines was used to cluster the development of clozapine concentrations over time. Quantile splines are a flexible, non-parametric regression technique that models specific percentiles of the outcome distribution rather than just its mean.^38^ Such a modeling technique allows for a detailed understanding of how different parts of the clozapine concentration distribution evolve and captures heterogeneous patterns across individuals.^38^ In total, 20 models were estimated, based on 5 or 7 quantile splines link functions and using 1-5 latent classes in both sexes. The final model selection was based on the lowest Bayesian information criterion (BIC) (see Appendix). Following LCGA, individual cluster assignments were extracted. To assess differences between selected trajectory classes across sexes, a linear mixed-effects model (LMEM) was applied. The model included age in weeks, class assignment*,* and their interaction as fixed effects to evaluate how clozapine levels varied between trajectory classes for men and women across all age ranges. A random intercept for every individual was included to account for within-subject effects. Estimated marginal means (EMMs) were computed, and pairwise comparisons between the trajectory classes were conducted to evaluate significant differences in clozapine concentrations. Statistical analyses were performed in R (version 4.4.2) via Rstudio (version 2024.12.0),^39^ and the LCGA was performed using the lcmm package (version 2.1.0).^40^^,^^41^

## Results

A total of 982 patients aged 40-60 years were included in the analyses, comprising 720 men (73.3%) and 262 women (26.7%). There were 17 104 clozapine blood level measurements with 13 271 (77.6%) observations from men and 3833 (22.4%) from women. Clozapine blood levels were on average, higher than the minimum therapeutic threshold of 350 μg/L, but were slightly below the advised guideline reference values of 400-700 μg/L^18^^,^^19^ with mean levels of 389 μg/L for women and 376 μg/L for men aged 40-60 years.

### LCGA Trajectories in Women

**Figure 1:**
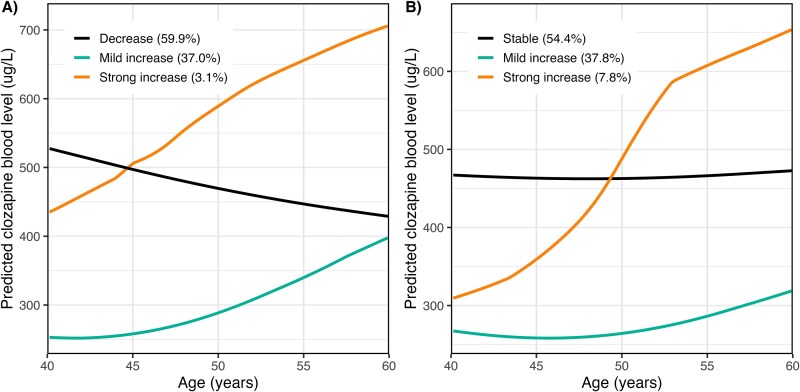
Observed Trajectories of Clozapine Blood Levels in (A) Women and (B) Men by Latent Class Growth Analysis

The optimal LCGA model with lowest BIC value (Figure A1) for women consisted of 3 clusters with 7-quantile splines. The majority of women (*n* = 157, 59.9%) showed decreasing clozapine levels from a mean of 520-400 μg/L over 20 years, as shown in Fig. 1A (*Decrease* class). The *Mild increase* class comprised 37.02% (*n* = 97) of the sample and showed an increase in clozapine levels starting at 250 μg/L from the age of 45 to an average of 400 μg/L over 15 years. The *Strong increase* class consisted of 3.05% (*n* = 8) women, had a higher baseline starting point compared to the *Decrease* class trajectory, and showed a further increase in clozapine levels to around 700 μg/L for women around 60 years. The average class probabilities for the model were around 78%, indicating good model fit and classification accuracy among women.

**Table 1:** Summary Statistics for Age, Measurement Count, and Clozapine Concentrations by Sex

	**Men (*n* = 720)**	**Women (*n* = 262)**	**Total (*n* = 982)**
Age at first measurement			
Mean (SD)	47.1 (6.01)	48.6 (5.82)	47.5 (5.99)
Median [Min, Max]	46.0 [40.0, 60.0]	49.0 [40.0, 60.0]	47.0 [40.0, 60.0]
Mean age across measurements			
Mean (SD)	48.7 (6.08)	50.3 (5.76)	49.1 (6.03)
Median [Min, Max]	47.9 [40.0, 60.0]	50.4 [40.0, 60.0]	48.8 [40.0, 60.0]
Number of measurements			
Mean (SD)	18.4 (17.2)	14.6 (12.0)	17.4 (16.1)
Median [Min, Max]	12.0 [4.00, 121]	10.5 [4.00, 80.0]	12.0 [4.00, 121]
Mean clozapine conc. (μg/L)			
Mean (SD)	371 (157)	389 (154)	376 (156)
Median [Min, Max]	384 [20.7, 809]	400 [47.8, 740]	389 [20.7, 809]

### LCGA Trajectories in Men

The optimal LCGA model with lowest BIC values (Figure A2) for men also consisted of 3 classes with 7-quantile splines. The *Stable* cluster included most men with 54.44% (*n* = 392) of the sample and showed relatively stable clozapine levels of on average 460 μg/L over time. The *Mild increase* cluster, which accounted for 37.78% (*n* = 272) of the sample, showed a more moderate upward trend in clozapine levels, starting around 50 years and increasing from 270 to 330 μg/L at 60 years (Figure 1B). The *Strong increase* cluster included 7.78% (*n* = 56) of the sample and exhibited the steepest increase in clozapine levels, starting at 300 μg/L and rising to approximately 600 μg/L over 20 years. The average class probabilities for the model were around 77%, indicating that 77% of the men were assigned to the most correct classes.

### Comparing Clozapine Concentrations Between Stable Men and Decreasing Women Trajectories

**Figure 2:**
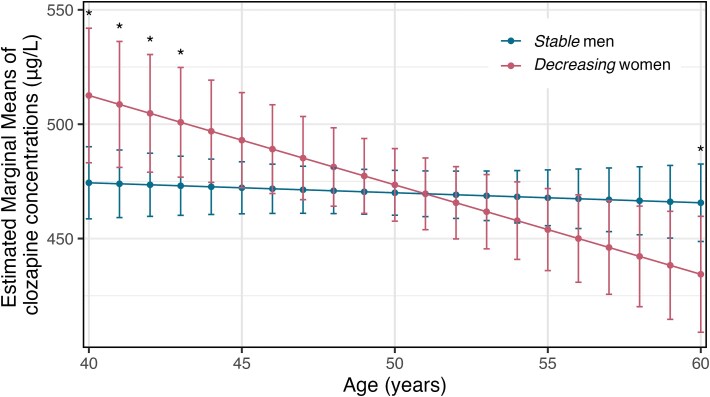
Estimated Marginal Means of Clozapine Concentrations (μg/L) by Age and Trajectory Group

LMEM was conducted to compare whether and to which extent clozapine concentrations differed between men in the *Stable* trajectory and women in the *Decreasing* trajectory. These trajectories were selected as they were notably different in the LCGA analyses of men and women*,* EMMs for clozapine concentrations were calculated for men and women at each age from 40 to 60 years (Table A1). The LMEM showed a significant effect of group on clozapine concentrations, with women in the decreasing group showing higher clozapine levels than men in the stable group (estimate = 177.03, *t* = 2.62, *P* < .01). The interaction between age and sex was significant, indicating that the difference between men and women changed over time (estimate = −0.067, *t* = −2.63, *P* < .01) (Figure 2). At age 40, women had significantly higher concentrations than men (mean difference = 38.17 μg/L, *t* = 2.25, *P* = .025). Similar significant differences were found at age 41 (mean difference = 34.69 μg/L, *t* = 2.18, *P* = .030), age 42 (mean difference = 31.22 μg/L, *t* = 2.10, *P* = .036), and at age 43 (mean difference = 27.75 μg/L, *t* = 2.00, *P* = .046). However, as age increased, this pattern reversed (Figure 2), with no significant differences between the stable men and decreasing women between ages 44 and 59 (all *P*-values > .05, Table A1). By age 60, the mean difference indicated that women had significantly lower concentrations than men (mean difference = −31.26 μg/L, *t* = −2.01, *P* = .044).

## Discussion

Our study is the first to investigate the longitudinal trajectories of clozapine blood concentrations across the perimenopausal ages of 40-60 years and compare these patterns between sexes. Our objective was to investigate whether clozapine concentrations decrease in women during perimenopause differently than in age-matched men. Our results indicated that most women showed a steady decline in clozapine concentrations between the ages of 40 and 60 years, while men, in contrast, maintained stable concentrations of clozapine over the same age span. Between ages 40 and 43 women had around 30 μg/L higher concentrations than men, while at age 60, this effect is reversed, and women had around 30 μg/L lower concentrations compared to men. These sex-specific differences support the hypothesis that estrogen-mediated inhibition of CYP1A2 and associated downregulation of the enzyme’s expression contributes to elevated clozapine concentrations in premenopausal women compared to age-matched men, an effect that diminishes and ultimately disappears with declining estrogen levels. A decline in clozapine levels could have consequences for treatment efficacy in menopausal women, especially during this life phase that is already associated with higher relapse risk.^42^^,^^43^

Our finding that women showed higher clozapine levels than men when aged in their early forties aligns with previous research showing higher average blood concentrations of clozapine in premenopausal women compared to men. Jönsson et al. found that dose-adjusted serum clozapine concentrations were significantly higher in women than men.^14^ Tang et al. observed higher mean clozapine levels in women, but they did not detect an age-related effect, possibly because their age groups were not stratified by sex.^44^ Similarly, Castberg et al. reported that women generally have 20%-30% higher clozapine dose-adjusted concentrations than men.^13^ These differences were observed in a mixed group of women of both pre- and postmenopausal age.^13^^,^^45^ A recent study performed in China reported that men need higher clozapine doses than women to reach therapeutic blood levels and in patients below age 44, but did not investigate sex-by-age interaction.^46^ Our work provides more detailed information and shows that higher blood levels are only seen in premenopausal women, yet not in older women. This information can help to refine guidelines for clozapine dosing, as it is a step toward personalized medicine.

We further showed that a significant subset of participants, 37.8% of men and 37% of women, exhibited mildly increasing clozapine levels over 20 years. In both sexes, these trajectories started at around age 45. In 3% of women and 8% of men, we also showed trajectories with a *Strong Increase* starting from a higher baseline concentration at and from an earlier age onwards. Several factors may contribute to these upward trends, including the age-related declines in renal clearance,^13^^,^^47^ higher rates of chronic kidney disease occurring in patients with schizophrenia spectrum disorders,^48^^,^^49^ or decreasing renal clearance due to co-medication with lithium, among individuals with schizoaffective disorder.^34^^,^^35^ Reducing or stopping smoking, a factor known to influence clozapine metabolism,^50–53^ could also partially account for these patterns. Dose increase could also be a factor, but steady dose increases over more than ten years are relatively rare.^44^

### Limitations

Our study has several limitations. First, we did not have access to participants’ daily clozapine prescribed doses to calculate dose-corrected concentrations. Without dosage, administration time, or treatment duration, we could not determine whether steady-state concentrations had been reached, nor assess steady-state interactions.^44^^,^^54^ Other limitations include the lack of data on ethnicity, smoking status, concomitant medications affecting CYP1A2 activity, exogenous hormone use (ie, hormonal contraceptives or hormone replacement therapy), substance use, treatment compliance, and diagnosis, all of which can affect clozapine blood concentrations.^13^^,^^15^^,^^20^^,^^43^^,^^45^^,^^47–50^^,^^55–57^ Given the longitudinal within-subject design and large sample sizes, we expect that these factors had only a limited effect on the latent growth class analyses. Finally, without the exact date of menopause, we could not directly relate the changes in clozapine levels to menopausal status. Despite these limitations, our study provides valuable insights into clozapine blood concentration trajectories, which can be further refined by future studies with more detailed clinical and demographic data.

## Conclusion

In conclusion, our study showed that most women experienced a decline in blood clozapine levels over the menopausal transition, while most men showed stable clozapine concentrations between 40 and 60 years. At age 40, average clozapine concentrations were some 30 μg/L higher in women compared to men, whereas by age 60, female clozapine levels were on average 30 μg/L lower than in men. Our findings highlight the changes in clozapine blood levels in women as they reach the ages of menopausal transition. Lower estrogen levels after menopause may reduce clozapine efficacy and increase the risk of psychotic relapse in an already vulnerable life phase. As many drugs have CYP1A2 as their main metabolite, an effect of menopause on blood levels can be expected for other medications as well.

## Supplementary Material

Appendix-Manuscript_sbaf186

## References

[1] Bosch EL, IEC S, Touw DJ. The influence of female sex and estrogens on drug pharmacokinetics: what is the evidence? *Expert Opin Drug Metab Toxicol*. 2025:637-647. 10.1080/17425255.2025.2481891 40109018

[2] Chadid S, Barber JR, Rohrmann S, et al. Age-specific serum total and free estradiol concentrations in healthy men in US nationally representative samples. *J Endocr Soc*. 2019;3:1825–1836. 10.1210/js.2019-00178 31555753 PMC6749840

[3] Richardson H, Ho V, Pasquet R, et al. Baseline estrogen levels in postmenopausal women participating in the MAP.3 breast cancer chemoprevention trial. *Menopause*. 2020;27:693–700. 10.1097/GME.0000000000001568 32433262 PMC7469568

[4] Radolph JF Jr, Sowers M, Bondarenko I, et al. The relationship of longitudinal change in reproductive hormones and vasomotor symptoms during the menopausal transition. *J Clin Endocrinol Metabolsm*. 2005;90:6106–6112. 10.1210/jc.2005-1374 16144949

[5] Sommer IE, Brand BA, Stuijt CCM, Touw DJ. Sex differences need to be considered when treating women with psychotropic drugs. *World Psychiatry*. 2024;23:151–152. 10.1002/wps.21155 38214636 PMC10785976

[6] Schoretsanitis G, Deligiannidis KM, Paulzen M, Spina E, de Leon J. Drug-drug interactions between psychotropic medications and oral contraceptives. *Expert Opin Drug Metab Toxicol*. 2022;18:395–411. 10.1080/17425255.2022.2106214 35876180

[7] Xie C, Pogribna M, Word B, Lyn-Cook L, Lyn-Cook BD, Hammons GJ. In vitro analysis of factors influencing *CYP1A2* expression as potential determinants of interindividual variation. *Pharmacol Res Perspect*. 2017;5:e00299. 10.1002/prp2.299 28357125 PMC5368963

[8] Mwinyi J, Cavaco I, Pedersen RS, et al. Regulation of CYP2C19 expression by estrogen receptor α: implications for estrogen-dependent inhibition of drug metabolism. *Mol Pharmacol*. 2010;78:886–894. 10.1124/mol.110.065540 20675569

[9] Edlinger M, Brettbacher S, Schurr T, Yalcin-Siedentopf N, Hofer A. No gender differences in the pharmacological emergency treatment of schizophrenia: results of a 21-year observation. *Int Clin Pharmacol*. 2024;39:36–41. 10.1097/YIC.0000000000000495 37555960

[10] Keepers GA, Fochtmann LJ, Anzia JM, et al. The American Psychiatric Association practice guideline for the treatment of patients with schizophrenia. *Am J Psychiatry*. 2020;177:868–872. 10.1176/appi.ajp.2020.177901 32867516

[11] Ventriglio A, Ricci F, Magnifico G, et al. Psychosocial interventions in schizophrenia: focus on guidelines. *Int J Soc Psychiatry*. 2020;66:735–747. 10.1177/0020764020934827 32597274

[12] Bachmann CJ, Aagaard L, Bernardo M, et al. International trends in clozapine use: a study in 17 countries. *Acta Psychiatr Scand*. 2017;136:37–51. 10.1111/acps.12742 28502099

[13] Castberg I, Westin AA, Skogvoll E, Spigset O. Effects of age and gender on the serum levels of clozapine, olanzapine, risperidone, and quetiapine. *Acta Psychiatr Scand*. 2017;136:455–464. 10.1111/acps.12794 28865402

[14] Jönsson AK, Spigset O, Reis M. A compilation of serum concentrations of 12 antipsychotic drugs in a therapeutic drug monitoring setting. *Ther Drug Monit*. 2019;41:348–356. 10.1097/FTD.0000000000000585 31025986 PMC6553956

[15] Chang YC, Tseng YT, Jann MW, Lane HY, Lin SK, Wen-Ho C. Effects of gender and age on plasma levels of clozapine and its metabolites: analyzed by critical statistics. *J Clin Psychiatry*. 1999;60:36–40. 10.4088/jcp.v60n0108 10074876

[16] Brand BA, Haveman YRA, De Beer F, De Boer JN, Dazzan P, Sommer IEC. Antipsychotic medication for women with schizophrenia spectrum disorders. *Psychol Med*. 2022;52:649–663. 10.1017/S0033291721004591 34763737 PMC8961338

[17] Scandlyn MJ, Stuart EC, Rosengren RJ. Sex-specific differences in CYP450 isoforms in humans. *Expert Opin Drug Metab Toxicol*. 2008;4:413–424. 10.1517/17425255.4.4.413 18524030

[18] Hiemke C, Bergemann N, Clement H, et al. Consensus guidelines for therapeutic drug monitoring in Neuropsychopharmacology: update 2017. *Pharmacopsychiatry*. 2018;51:9–62. 10.1055/s-0043-116492 28910830

[19] Schoretsanitis G, Kane JM, Correll CU, et al. Blood levels to optimize antipsychotic treatment in clinical practice: a joint consensus statement of the American Society of Clinical Psychopharmacology and the therapeutic drug monitoring task force of the Arbeitsgemeinschaft für Neuropsychopharmakologie und Pharmakopsychiatrie. *J Clin Psychiatry*. 2020;81:19cs13169. 10.4088/JCP.19cs13169 32433836

[20] Shlomi Polachek I, Manor A, Baumfeld Y, et al. Sex differences in psychiatric hospitalizations of individuals with psychotic disorders. *J Nerv Ment Dis*. 2017;205:313–317. 10.1097/NMD.0000000000000645 28129306

[21] Sommer IE, Brand BA, Gangadin S, Tanskanen A, Tiihonen J, Taipale H. Women with schizophrenia-Spectrum disorders after menopause: a vulnerable group for relapse. *Schizophr Bull*. 2023;49:136–143. 10.1093/schbul/sbac139 36198044 PMC9810004

[22] Arar MA, Erbil N. The effect of menopausal symptoms on women’s daily life activities. *Menopausal Rev*. 2023;22:6–15. 10.5114/pm.2023.126436 PMC10189673 37206677

[23] D’Angelo S, Bevilacqua G, Hammond J, Zaballa E, Dennison EM, Walker-Bone K. Impact of menopausal symptoms on work: findings from women in the health and employment after fifty (HEAF) study. *Int J Environ Res Public Health*. 2022;20:295. 10.3390/ijerph20010295 36612616 PMC9819903

[24] Tiwari S, Prasad R, Wanjari MB, Sharma R. Understanding the impact of menopause on women with schizophrenia-spectrum disorders: a comprehensive review. *Cureus*. 2023;15:e37979. 10.7759/cureus.37979 PMC10202668 37223185

[25] Hess R, Thurston RC, Hays RD, et al. The impact of menopause on health-related quality of life: results from the STRIDE longitudinal study. *Qual Life Res*. 2012;21:535–544. 10.1007/s11136-011-9959-7 21755412 PMC3252474

[26] Lewitus VJ, Kim J, Blackwell KT. Sex and estradiol effects in the rodent dorsal striatum. *Eur J Neurosci*. 2024;60:6962–6986. 10.1111/ejn.16607 39573926 PMC11647445

[27] Tveito M, Høiseth G, Haslemo T, Molden E, Smith RL. Impact of age and gender on paliperidone exposure in patients after administration of long-acting injectable formulations—an observational study using blood samples from 1223 patients. *Eur J Clin Pharmacol*. 2021;77:1201–1208. 10.1007/s00228-021-03114-z 33616704

[28] Edinoff AN, Fort JM, Woo JJ, et al. Selective serotonin reuptake inhibitors and clozapine: clinically relevant interactions and considerations. *Neurol Int*. 2021;13:445–463. 10.3390/neurolint13030044 34564289 PMC8482107

[29] Zorginstituut Nederland. Venlafaxine. Farmacotherapeutischcompass. [cited 2025 Feb 1] https://www.farmacotherapeutischkompas.nl/bladeren/preparaatteksten/v/venlafaxine

[30] Dodge DL . Increase in clozapine levels following the introduction of venlafaxine: a case report. *Bull Clin Psychopharmacol*. 2012;22:112–112. 10.5455/bcp.20110815070413

[31] Jeppesen U, Gram LF, Vistisen K, Loft S, Poulsen HE, Brøsen K. Dose-dependent inhibition of CYP1A2, CYP2C19 and CYP2D6 by citalopram, fluoxetine, fluvoxamine and paroxetine. *Eur J Clin Pharmacol*. 1996;51:73–78. 10.1007/s002280050163 8880055

[32] Deodhar M, Rihani SBA, Darakjian L, Turgeon J, Michaud V. Assessing the mechanism of fluoxetine-mediated CYP2D6 inhibition. *Pharmaceutics*. 2021;13:148. 10.3390/pharmaceutics13020148 33498694 PMC7912198

[33] Tiihonen J, Vartiainen H, Hakola P. Carbamazepine-induced changes in plasma levels of neuroleptics. *Pharmacopsychiatry*. 1995;28:26–28. 10.1055/s-2007-979584 7746842

[34] Ikawa K, Imura T, Nakamura S, et al. Effects of antiepileptic drugs, carbamazepine and phenytoin, on plasma concentrations of clozapine and its *N*-desmethyl and *N* -oxide metabolites in Japanese patients with schizophrenia. *Neuropsychopharmacol Rep*. 2025;45:e70035. 10.1002/npr2.70035 40567179 PMC12198689

[35] Lutz JD, VandenBrink BM, Babu KN, Nelson WL, Kunze KL, Isoherranen N. Stereoselective inhibition of CYP2C19 and CYP3A4 by fluoxetine and its metabolite: implications for risk assessment of multiple time-dependent inhibitor systems. *Drug Metab Dispos*. 2013;41:2056–2065. 10.1124/dmd.113.052639 23785064 PMC3834134

[36] Mian P, Somers M, Berg MT, Cahn W, Wilting I, Schaik RV. High levels of several antipsychotics and antidepressants due to a pharmacogenetic cause: a case report. *Pharmacogenomics*. 2019;20:567–570. 10.2217/pgs-2019-0037 31190622

[37] Teuling ND, Pauws S, Heuvel E v d. Clustering of longitudinal data: a tutorial on a variety of approaches. arXiv. 10.48550/arXiv.2111.05469 November 10, 2021, preprint: not peer reviewed

[38] Koenker R . Quantile Regression. Cambridge University Press; 2005. 10.1017/CBO9780511754098

[39] R Core Team . R: A Language and Environment for Statistical Computing. Vienna (Austria): R Foundation for Statistical Computing; 2020.

[40] Proust-Lima C, Phillips V, Diakite A, Liquet B. lcmm: Extended Mixed Models Using Latent Classes and Latent Processes. 2025 [cited 2025 Jan 1]. https://cran.r-project.org/package=lcmm

[41] Proust-Lima C, Phillips V, Liquet B. Estimation of extended mixed models using latent classes and latent processes: the R package lcmm. *J Stat Softw*. 2017;78:1–56. 10.18637/jss.v078.i02

[42] Siskind D, Sharma M, Pawar M, et al. Clozapine levels as a predictor for therapeutic response: a systematic review and meta-analysis. *Acta Psychiatr Scand*. 2021;144:422–432. 10.1111/acps.13361 34374073

[43] Schoretsanitis G, Kane JM, Ruan CJ, Spina E, Hiemke C, De Leon J. A comprehensive review of the clinical utility of and a combined analysis of the clozapine/norclozapine ratio in therapeutic drug monitoring for adult patients. *Expert Rev Clin Pharmacol*. 2019;12:603–621. 10.1080/17512433.2019.1617695 31075044

[44] Tang Y, Mao P, Li F, et al. Gender, age, smoking behaviour and plasma clozapine concentrations in 193 Chinese inpatients with schizophrenia. *Br J Clin Pharmacol*. 2007;64:49–56. 10.1111/j.1365-2125.2007.02852.x 17298477 PMC2000616

[45] González-Rodríguez A, Monreal JA, Seeman MV. The effect of menopause on antipsychotic response. *Brain Sci*. 2022;12:1342. 10.3390/brainsci12101342 36291276 PMC9599119

[46] Ding J, Zhang S, Li L, et al. Daily dosing frequency as a determinant of clozapine concentration-to-dose ratio: data from a therapeutic drug monitoring service (2019–2022). *Clin Chim Acta*. 2025;566:120064. 10.1016/j.cca.2024.120064 39613021

[47] Ismail Z, Wessels AM, Uchida H, et al. Age and sex impact clozapine plasma concentrations in inpatients and outpatients with schizophrenia. *Am J Geriatr Psychiatry*. 2012;20:53–60. 10.1097/JGP.0b013e3182118318 21422906

[48] Tzur Bitan D, Krieger I, Berkovitch A, Comaneshter D, Cohen A. Chronic kidney disease in adults with schizophrenia: a nationwide population-based study. *Gen Hosp Psychiatry*. 2019;58:1–6. 10.1016/j.genhosppsych.2019.01.007 30807892

[49] Carswell C, Cogley C, Bramham K, Chilcot J, Noble H, Siddiqi N. Chronic kidney disease and severe mental illness: a scoping review. *J Nephrol*. 2023;36:1519–1547. 10.1007/s40620-023-01599-8 37029882 PMC10393892

[50] Rostami-Hodjegan A, Amin AM, Spencer EP, Lennard MS, Tucker GT, Flangan RJ. Influence of dose, cigarette smoking, age, sex, and metabolic activity on plasma clozapine concentrations: a predictive model and nomograms to aid clozapine dose adjustment and to assess compliance in individual patients. *J Clin Psychopharmacol*. 2004;24:70–78. 10.1097/01.jcp.0000106221.36344.4d 14709950

[51] Wagner E, McMahon L, Falkai P, Hasan A, Siskind D. Impact of smoking behavior on clozapine blood levels – a systematic review and meta-analysis. *Acta Psychiatr Scand*. 2020;142:456–466. 10.1111/acps.13228 32869278

[52] Bozikas VP, Papakosta M, Niopas I, Karavatos A, Mirtsou-Fidani V. Smoking impact on CYP1A2 activity in a group of patients with schizophrenia. *Eur Neuropsychopharmacol*. 2004;14:39–44. 10.1016/S0924-977X(03)00061-0 14659985

[53] Tsuda Y, Saruwatari J, Yasui-Furukori N. Meta-analysis: the effects of smoking on the disposition of two commonly used antipsychotic agents, olanzapine and clozapine. *BMJ Open*. 2014;4:e004216. 10.1136/bmjopen-2013-004216 PMC3948577 24595134

[54] Perry PJ, Bever KA, Arndt S, Combs MD. Relationship between patient variables and plasma clozapine concentrations: a dosing nomogram. *Biol Psychiatry*. 1998;44:733–738. 10.1016/S0006-3223(97)00531-3 9798077

[55] Fransson F, Werneke U, Öhlund L, Jonsson PA, Ott M. Kidney function decline improves after lithium discontinuation. *J Intern Med*. 2025;297:289–299. 10.1111/joim.20054 39829336 PMC11846072

[56] Small JG, Klapper MH, Malloy FW, Steadman TM. Tolerability and efficacy of clozapine combined with lithium in schizophrenia and schizoaffective disorder. *J Clin Psychopharmacol*. 2003;23:223–228. 10.1097/01.jcp.0000084026.22282.5f 12826983

[57] González-Díaz J, Lozano Lemes L, Durante Niño M, Zamora D, Bioque M. Prescripción ambulatoria de clozapina en Colombia: factores relacionados con el uso de dosis inferiores a 100 mg/día [Outpatient prescription of clozapine in Colombia: factors related to the use of doses lower than 100 mg/day]. *Vertex*. 2024;35:82–85. 10.53680/vertex.v35i164.550 39024483

