# Supplementary material for: Trajectories of Clozapine Concentrations in Women Across Menopausal Age
Source: Schizophr Bull. 2025 Oct 28;52(2):sbaf186. doi: 10.1093/schbul/sbaf186 (PMC13005174; doi:10.1093/schbul/sbaf186)
Supplement: Appendix-Manuscript_sbaf186 [file appendix-manuscript_sbaf186.docx]

**Appendix**

Figure A1. Comparison of BIC Values Across Methods and Latent Classes for Women Models


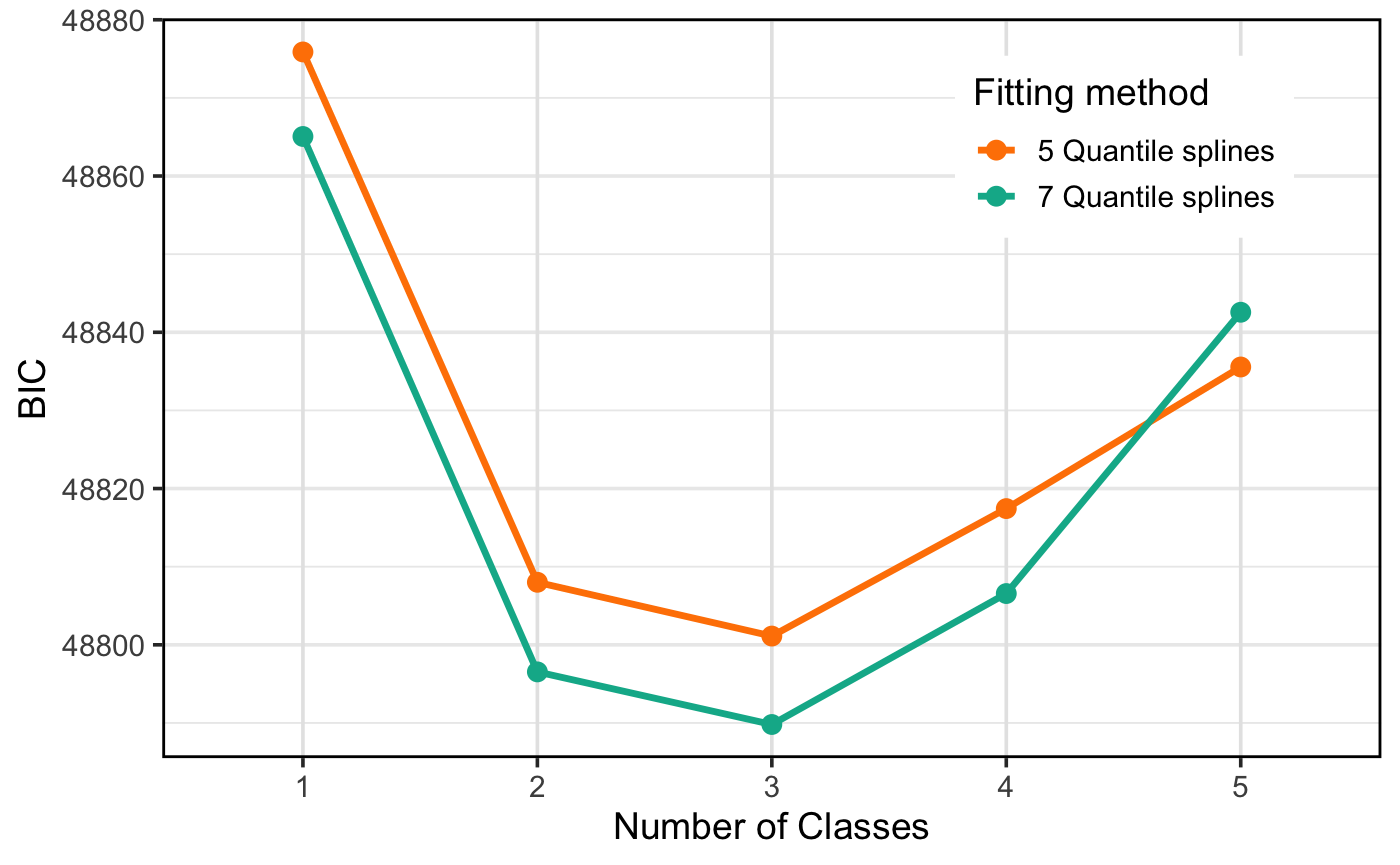


Figure A2. Comparison of BIC Values Across Methods and Latent Classes for Men Models
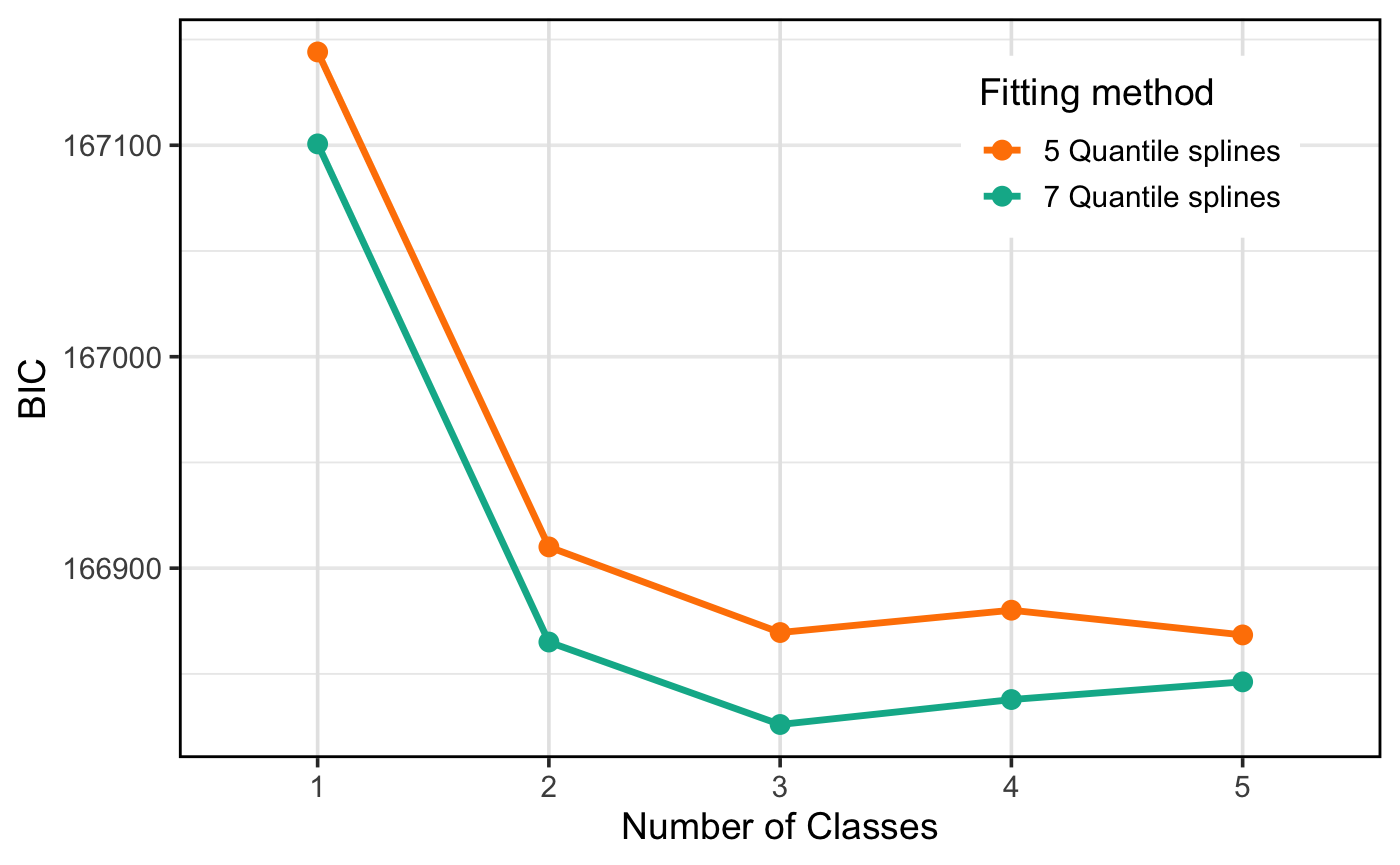


Table A1. Pairwise Comparison of Estimated Marginal Means for the Contrast *Decreasing* women group – *Stable* men group of Clozapine Concentrations by Age

| Age | estimate | Stand. error | df | t ratio | p-value |
| --- | --- | --- | --- | --- | --- |
| **40** | **38.17** | **17.00** | **843.81** | **2.25** | **0.025** |
| **41** | **34.70** | **15.91** | **818.99** | **2.18** | **0.030** |
| **42** | **31.22** | **14.87** | **791.19** | **2.10** | **0.036** |
| **43** | **27.75** | **13.88** | **760.40** | **2.00** | **0.046** |
| 44 | 24.28 | 12.94 | 726.97 | 1.88 | 0.061 |
| 45 | 20.81 | 12.08 | 691.64 | 1.72 | 0.085 |
| 46 | 17.34 | 11.30 | 655.81 | 1.53 | 0.125 |
| 47 | 13.87 | 10.63 | 621.54 | 1.30 | 0.193 |
| 48 | 10.40 | 10.10 | 591.54 | 1.03 | 0.304 |
| 49 | 6.92 | 9.71 | 568.79 | 0.71 | 0.476 |
| 50 | 3.45 | 9.49 | 556.01 | 0.36 | 0.716 |
| 51 | -0.02 | 9.46 | 554.95 | 0.00 | 0.998 |
| 52 | -3.49 | 9.61 | 565.88 | -0.36 | 0.717 |
| 53 | -6.96 | 9.93 | 587.51 | -0.70 | 0.484 |
| 54 | -10.43 | 10.42 | 617.32 | -1.00 | 0.317 |
| 55 | -13.91 | 11.04 | 652.28 | -1.26 | 0.208 |
| 56 | -17.38 | 11.78 | 689.44 | -1.48 | 0.141 |
| 57 | -20.85 | 12.61 | 726.46 | -1.65 | 0.099 |
| 58 | -24.32 | 13.52 | 761.70 | -1.80 | 0.072 |
| 59 | -27.79 | 14.50 | 794.21 | -1.92 | 0.056 |
| **60** | **-31.26** | **15.52** | **823.56** | **-2.01** | **0.044** |

**Note: significant results have been highlighted in bold**
